# Healthy Employments

**Published:** 1877-01

**Authors:** 


### HEALTH OF EMPLOYMENTS.

The following instructive table was prepared by direction of the Massachusetts Legislature, by which it appears that the average age of

| | Years. | | Years. |
|---|---|---|---|
| Gentlemen is . . . . . | 68 | Agriculturalists . . . . | 64 |
| Judges . . . . . . . | 65 | Bakers . . . . . . . . | 43 |
| Farmers . . . . . . . | 64 | Bank Officers . . . . | 64 |
| Bank Officers . . . . | 64 | Blacksmiths . . . . . . | 51 |
| Coopers . . . . . . . | 58 | Butchers . . . . . . . | 50 |
| Public Officers . . . . | 57 | Calico Printers . . . . | 51 |
| Clergymen . . . . . | 56 | Carpenters . . . . . . . | 49 |
| Shipwrights . . . . . | 55 | Clerks . . . . . . . . | 34 |
| Hatters . . . . . . . . | 54 | Clergymen . . . . . . | 56 |
| Lawyers . . . . . . . | 54 | Coopers . . . . . . . | 58 |
| Ropemakers . . . . . | 54 | Editors . . . . . . . . | 40 |
| Blacksmiths . . . . . | 51 | Gentlemen . . . . . . | 68 |
| Merchants . . . . . . | 51 | Hatters . . . . . . . . | 54 |
| Calico Printers . . . . | 51 | Jewellers . . . . . . . | 44 |
| Physicians . . . . . . | 51 | Judges and Justices . . | 65 |
| Butchers . . . . . . . | 50 | Lawyers . . . . . . . | 54 |
| Carpenters . . . . . . | 49 | Machinists . . . . . . . | 38 |
| Masons . . . . . . . . | 48 | Manufacturers . . . . | 43 |
| Traders . . . . . . . . | 46 | Masons . . . . . . . . | 48 |
| Tailors . . . . . . . . | 44 | Mechanics . . . . . . . | 43 |
| Jewellers . . . . . . . | 44 | Merchants . . . . . . . | 51 |
| Manufacturers . . . . | 43 | Musicians . . . . . . . | 39 |
| Bakers . . . . . . . . | 43 | Operatives . . . . . . . | 32 |
| Painters . . . . . . . | 43 | Painters . . . . . . . | 43 |
| Shoemakers . . . . . | 43 | Physicians . . . . . . . | 51 |
| Mechanics . . . . . . . | 43 | Printers . . . . . . . . | 38 |
| Editors . . . . . . . . | 40 | Public Officers . . . . | 57 |
| Musicians . . . . . . . | 39 | Ropemakers . . . . . . | 54 |
| Printers . . . . . . . | 38 | Shipwrights . . . . . . | 55 |
| Machinists . . . . . . . | 36 | Shoemakers . . . . . . | 43 |
| Teachers . . . . . . . | 34 | Tailors . . . . . . . . | 44 |
| Clerks . . . . . . . . | 34 | Teachers . . . . . . . | 34 |
| Operatives . . . . . | 32 | Traders . . . . . . . | 46 |

The most striking discrepancy in the above table is between the lifetime of a "gentleman"—that is, one who lives on his income—and a common laborer, who lives, in an expressive vulgarism, "from hand to mouth"—who lives upon the proceeds of each day's work. A "gentleman," with his surroundings, lives more than twice as long as the man, who if he does not find work today, must go without food, or go in debt to-morrow. A like result was given in our second volume, under the head of "Money a Medicine," as having been noticed in the report of M. Villerme, in France, where the average age of a thousand "prosperous" persons was forty-two years, while the average age of twenty thousand "poor" persons was twenty years—less than one half! about the same as in Massachusetts: showing, that in States, climes, and continents, as wide asunder in locality as in their civil politics, the same great general principles prevail in reference to the human body and mind, to wit, that *a mind at ease gives long life to the body.* On the same principles is it, that pensioned people and paupers, who feel that they are provided for, so often live to a good old age.

Next to the "gentleman" comes the salaried man, judges, justices, bank officers, and public officers—who can go to bed any night with the quiet assurance that they will not wake up paupers in the morning; that whatever betides, their salaries go on. In sad contrast with this are printers, machinists, clerks, and teachers, whose bread stops with their daily labor, or depends on the caprice of another. The broad fact thus comes out, that between dependence and independence there is literally an age, a life-time, or, in Wall-street language, a difference of one hundred per cent: the difference in despotic France of twenty and forty-two; in democratic America, of thirty-two and sixty-eight; and let the weekly mortuary tables of New York reiterate the same lesson, when they show us that of the multitudes who are daily striving for bread, one out of every six dies from a disease of the brain or nerves; showing, that the next great destroyer to remorseless consumption is *worldly care!*—the greed of gold—the strife for bread!

For these things, the Bible at once presents a rational, a speedy, a certain remedy, and withal practicable, when it warns us against hasting to be rich; counselling us to be moderate in our ambition, and to be "TEMPERATE IN ALL THINGS."

---

"THE best student and the best scholar in our seminary is the healthiest man in it," said a young theologian to us the other day; "and thirty-five per cent of the young men are daily absent at roll-call—excuse, 'unwell.'"

